# Integrative Analysis of miRNA and mRNA Expression Profiles in Mammary Glands of Holstein Cows Artificially Infected with *Staphylococcus aureus*

**DOI:** 10.3390/pathogens10050506

**Published:** 2021-04-22

**Authors:** Xiaolong Wang, Yongliang Fan, Yifan He, Ziyin Han, Zaicheng Gong, Yalan Peng, Yining Meng, Yongjiang Mao, Zhangping Yang, Yi Yang

**Affiliations:** 1 College of Animal Science and Technology, Yangzhou University, Yangzhou 225009, China; wangxl@yzu.edu.cn (X.W.); dx120170088@yzu.edu.cn (Y.F.); mx120170661@yzu.edu.cn (Z.H.); cattle@yzu.edu.cn (Y.M.); yzp@yzu.edu.cn (Z.Y.); 2 Jiangsu Co-Innovation Center for the Prevention and Control of Important Animal Infectious Diseases and Zoonoses, College of Veterinary Medicine, Yangzhou University, Yangzhou 225009, China; 182002109@yzu.edu.cn (Y.H.); MZ120191119@yzu.edu.cn (Z.G.); MX120190742@yzu.edu.cn (Y.P.); MZ120201429@yzu.edu.cn (Y.M.); 3 International Corporation Laboratory of Agriculture and Agricultural Products Safety, Yangzhou 225009, China

**Keywords:** bovine mastitis, *Staphylococcus aureus*, differential expression microRNAs, differentially expressed genes, integrative analysis

## Abstract

*Staphylococcus aureus*- induced mastitis is one of the most intractable problems for the dairy industry, which causes loss of milk yield and early slaughter of cows worldwide. Few studies have used a comprehensive approach based on the integrative analysis of miRNA and mRNA expression profiles to explore molecular mechanism in bovine mastitis caused by *S. aureus*. In this study, *S. aureus* (A1, B1 and C1) and sterile phosphate buffered saline (PBS) (A2, B2 and C2) were introduced to different udder quarters of three individual cows, and transcriptome sequencing and microarrays were utilized to detected miRNA and gene expression in mammary glands from the challenged and control groups. A total of 77 differentially expressed microRNAs (DE miRNAs) and 1625 differentially expressed genes (DEGs) were identified. Gene Ontology (GO) annotation and Kyoto Encyclopedia of Genes and Genomes (KEGG) pathway analysis showed that multiple DEGs were enriched in significant terms and pathways associated with immunity and inflammation. Integrative analysis between DE miRNAs and DEGs proved that miR-664b, miR-23b-3p, miR-331-5p, miR-19b and miR-2431-3p were potential factors regulating the expression levels of *CD14 Molecule* (*CD14*), *G protein subunit gamma 2* (*GNG2*), *interleukin 17A* (*IL17A*), *collagen type IV alpha 1 chain* (*COL4A1*), *microtubule associated protein RP/EB family member 2* (*MAPRE2*), *member of RAS oncogene family* (*RAP1B*), *LDOC1 regulator of NFKB signaling* (*LDOC1*), *low-density lipoprotein receptor* (*LDLR*) and *S100 calcium binding protein A9* (*S100A9*) in bovine mastitis caused by *S. aureus*. These findings could enhance the understanding of the underlying immune response in bovine mammary glands against *S. aureus* infection and provide a useful foundation for future application of the miRNA–mRNA-based genetic regulatory network in the breeding cows resistant to *S. aureus*.

## 1. Introduction

Bovine mastitis compromises the health and welfare of dairy cattle, as well as decreases the quality and quantity of milk production, causing huge economic losses in the global dairy industry [1]. *Staphylococcus aureus* is a major etiological pathogen of bovine mastitis, especially subclinical mastitis, causing a persistent and chronic infection, and antibiotic therapies are largely ineffective [2,3,4]. The infectivity and antibiotic resistance of *S. aureus* and other causative agents make bovine mastitis more difficult to control, which is also a risk of public health [5,6,7,8,9]. By breeding dairy cattle resistance to udder diseases, the risk of mastitis may be reduced in the dairy cow population [10]. Therefore, the identification of specific genes related to mastitis susceptibility or resistance can provide a new way to control mastitis through genetic selection [11,12].

In recent years, numerous studies have shown that bovine mammary epithelial cells (BMECs) respond to the invasion of bacteria or bacterial products by altering the expression levels of several genes involved in inflammation and immunity in vitro [13,14,15]. However, one limitation of these studies is that the conclusions drawn at cellular levels are not necessarily consistent with those of individuals [16]. Although some transcriptome-wide association studies have been carried out on *S. aureus*-induced mastitis in vivo, these studies always analyzed the expression levels of mRNAs or microRNAs (miRNAs) separately [17,18,19,20,21]. Few studies used a comprehensive approach based on the integrative analysis of miRNA and mRNA expression profiles to improve the understanding of the underlying molecular mechanism of cow mastitis caused by *S. aureus*.

To investigate various interaction networks and regulatory modes of mRNAs and miRNAs, we constructed a *S. aureus*-type bovine mastitis model and integrated the analysis of miRNAs and mRNAs between the *S. aureus*-infected quarters and the control ones. These findings will provide new insights into the mechanism of *S. aureus*-induced cow mastitis.

## 2. Results

### 2.1. The Establishment of Bovine S. aureus-Induced Mastitis Model

Indicators of the three cows were measured and recorded after bacterial infection. At 48 h post inoculation, the dairy cattle suffered from obvious pain and had a drastic reduction (25.8% reduction in average) in milk yield. In addition, the temperature of the cows raised (1.7 °C in average), and their mammary glands and lymph nodes were swollen and hard. At the same time, the alteration of the biophysical properties of milk (grey–white color) was observed. There were significant increases of somatic cell count (SCC) of the milk from inoculated quarters (A1: 1,790,000/mL; B1: 1,920,000/mL; and C1: 2,080,000/mL), while those from the controls remained below 100,000/mL.

### 2.2. The Pathological Observation

**Figure 1:**
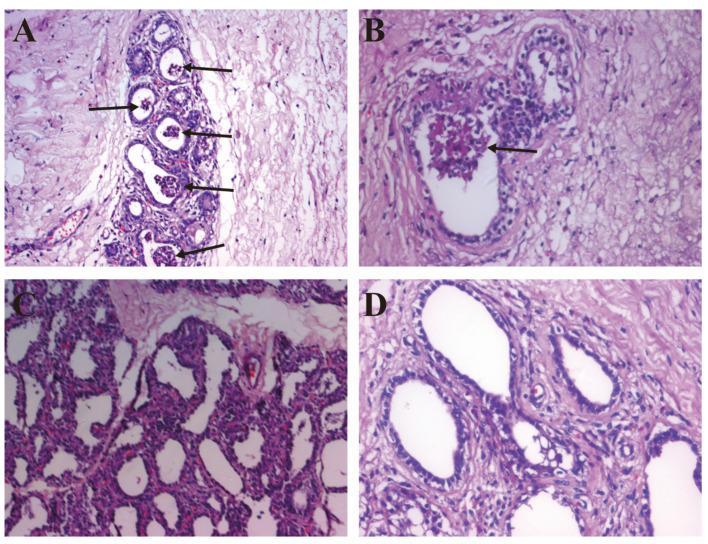
HE staining of mammary tissues. (**A**) Mammary tissues from the *S. aureus*-inoculated group with the infiltration of a large number of inflammatory cells, 200×. (**B**) Mammary tissues from the *S. aureus*-inoculated group with the infiltration of a large number of inflammatory cells, 400×. (**C**) Mammary tissues from the control group with an integrated structure, 200×. (**D**) Mammary tissues from the control group with an integrated structure, 400×. Arrowheads point to the mammary tissues with immune infiltrate.

Compared with the control group, the mammary epithelial cells in the *S. aureus*-inoculated group were loosely connected and had a lager intercellular space. A large number of inflammatory cells, including shed mammary epithelial cells, macrophages, neutrophils and lymphocytes, were clustered in the acini (Figure 1).

### 2.3. Differential Expressed miRNA Identification

**Figure 2:**
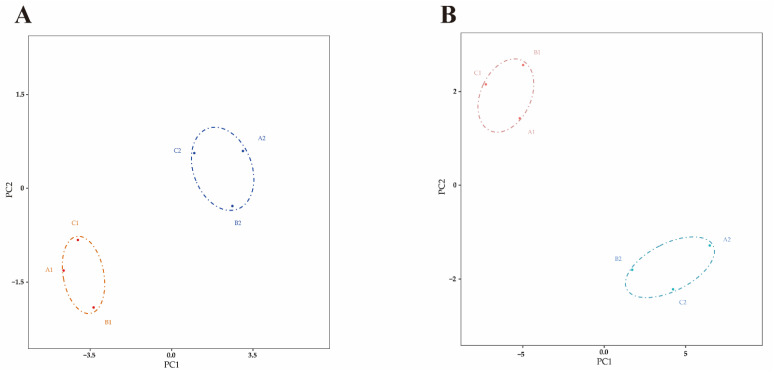
PCA analysis. (**A**) PCA analysis of miRNAs. (**B**) PCA analysis of mRNAs.

**Figure 3:**
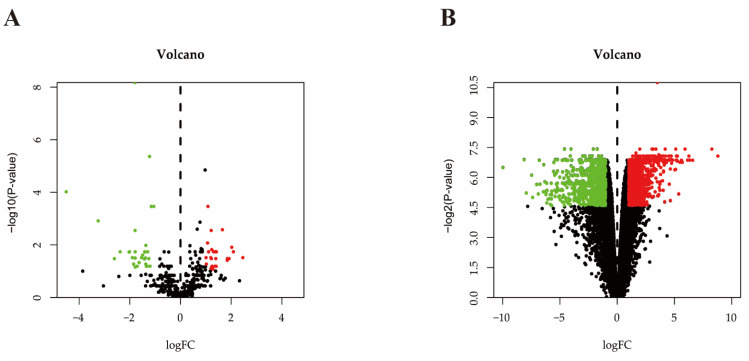
The volcano plots. (**A**) DE miRNAs in bovine mammary gland between the control group and *S. aureus*-inoculated group. The up-regulated and down-regulated miRNAs are shown in red and green dots, respectively, while the miRNAs with no significant difference in the two groups are shown in black dots. (**B**) DEGs in bovine mammary gland between the control group and *S. aureus*-inoculated group. The up-regulated and down-regulated mRNAs are indicated by red and green dots, respectively, while the mRNAs with no significant difference in the two groups are indicated by black dots.

A total of 21,293,853 and 18,588,177 raw reads were generated from the control and *S. aureus*-inoculated groups, respectively, by miRNA sequencing (Table S1). After raw reads were disposed, there were 20,847,000 and 18,504,775 clean reads for length distribution assessment. The assessment results revealed that the 78.76% and 71.79% of clean reads were 20–24 nucleotides in length in the two groups (Figure S1). Principal component analysis (PCA) showed the miRNAs in the challenged and control groups can be classified into different clusters, respectively, indicating sequencing data is qualified for further analysis (Figure 2A). A total of 77 DE miRNAs, including 30 up-regulated and 47 down-regulated miRNAs (*p* ≤ 0.05 and |log_2_FC| ≥ 1), were identified in the *S. aureus*-inoculated group, compared with control group (Figure 3A).

### 2.4. Differential Expressed mRNA Identification

The values of 2100 RIN and 28S/18S were between 7.5–8.9 and 1.3–2.1, respectively (Table S2), indicating that the RNA quality met the requirement and could be used for marker hybridization.

In this study, the CV values of all samples ranged from 3.389% to 4.821% (Table S3), indicating that the detection results of the microarray are reliable.

The PCA was also performed to evaluate the sample distribution. Two separate clusters were found, representing the *S. aureus* inoculation and control groups, respectively (Figure 2B). The transcriptional sequences of the same group were assembled in the same cluster, indicating that the main differences in the mRNA expression profiles occurred between different groups.

A total of 1030 up-regulated genes and 595 down-regulated genes (*p* ≤ 0.05 and |log_2_FC| ≥ 1) were identified in the *S. aureus* inoculation group versus control group (Figure 3B).

### 2.5. Interaction Analysis of the miRNAs and mRNAs

**Figure 4:**
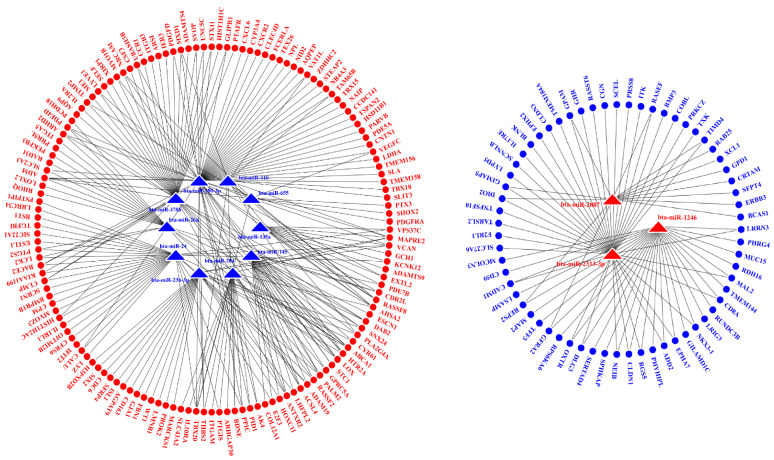
miRNA–mRNA interaction networks. **Red** and **blue** triangles represent up-regulated and down-regulated miRNA in the *S. aureus*-inoculated group, respectively. **Red** and **blue** circles represent up-regulated and down-regulated DEGs in the *S. aureus*-inoculated group, respectively.

Three up-regulated and ten down-regulated DE miRNAs (*p* ≤ 0.05 and |log_2_FC| ≥ 2) were selected for the miRNA–mRNA interactive analysis. Among all potential target genes predicted by TargetScan, 143 up-regulated and 63 down-regulated genes identified in this study were employed for the construction of miRNA–mRNA interaction networks (Figure 4).

Among the DE miRNAs and DEGs (*p* ≤ 0.05 and |log_2_FC| ≥ 2) employed for the interaction analysis, 76.92% (10/13) of the DE miRNAs and 16.50% (34/206) of the DEGs had been identified by previous studies [20,22,23,24,25,26,27,28,29,30,31].

### 2.6. Functional Analysis of Differentially Expressed Genes

**Figure 5:**
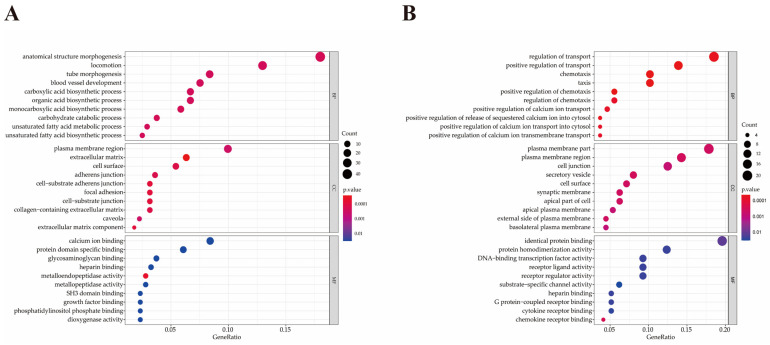
GO functional enrichment analysis of DEGs. (**A**) Top 10 significant biological process, cellular component and molecular function terms enriched by up-regulated DEGs. (**B**) Top 10 significant biological process, cellular component and molecular function terms enriched by down-regulated DEGs.

**Figure 6:**
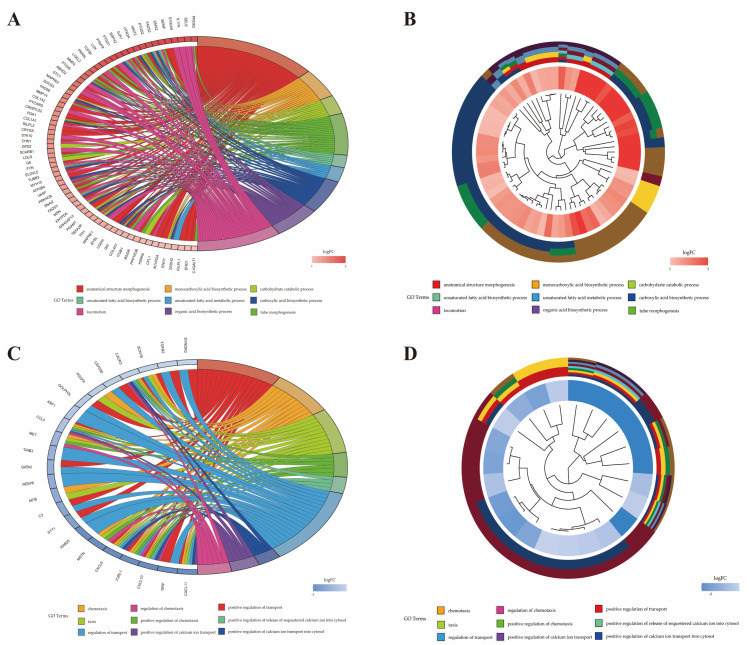
Features of DEGs enriched in top 9 significant GO terms. (**A**) Circos plots show overlapping and specific responses of up-regulated DEGs. (**B**) Circos plots summarize features of up-regulated DEGs. (**C**) Circos plots show overlapping and specific responses of down-regulated DEGs. (**D**) Circos plots summarize features of down-regulated DEGs.

The Gene Ontology (GO) annotation based on three categories (biological processes (BP), molecular functions (MF) and cellular component (CC)) was performed to explore biological functions of DEGs regulated by DE miRNAs, in which there were 721 up-regulated and 381 down-regulated genes. The 721 up-regulated genes were significantly enriched in 174 BP terms, 31 MF terms and 25 CC terms. Among them, 68 up-regulated genes of 19 terms were involved in inflammation and immune response (Table 1). The 381 down-regulated genes were significantly enriched in 199 BP terms, 23 MF terms and 37 CC terms. Among them, 21 down-regulated genes of 25 terms were involved in inflammation and immune response. Only the top 10 up-regulated and down-regulated terms in each category are listed in Figure 5. Features of DEGs enriched in the top 9 significant GO terms are shown in Figure 6.

**Figure 7:**
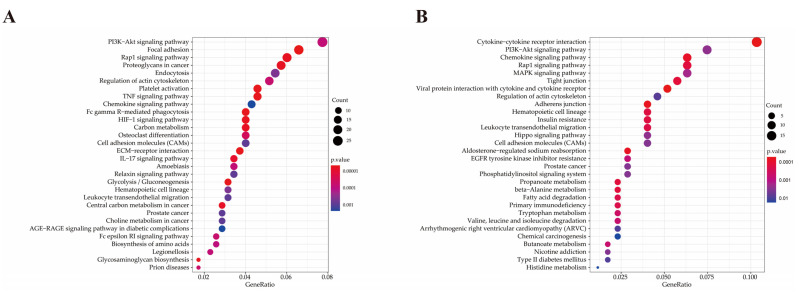
KEGG pathway analysis of DEGs. (**A**) Scatter plots of the top 30 significant enriched KEGG pathways of up-regulated DEGs. (**B**) Scatter plots of the top 30 significant enriched KEGG pathways of down-regulated DEGs.

**Figure 8:**
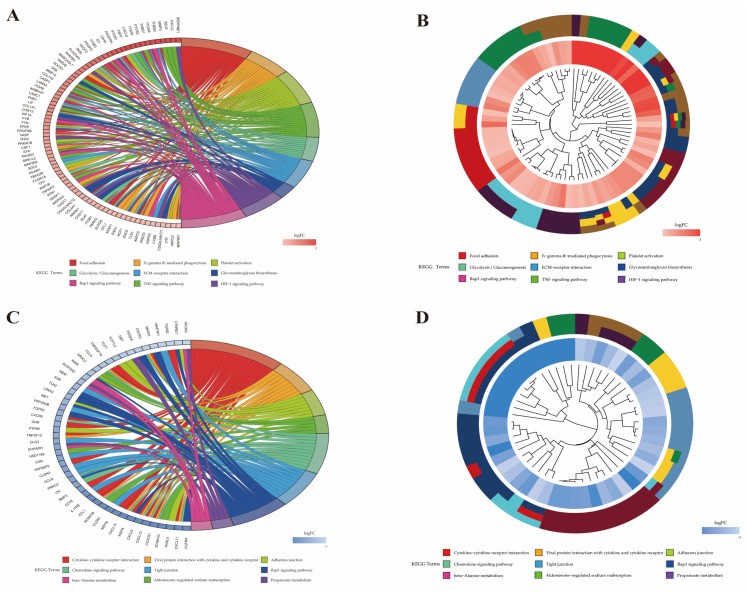
Features of DEGs enriched in the top 30 significant KEGG pathways. (**A**) Circos plots show overlapping and specific responses of up-regulated DEGs. (**B**) Circos plots summarize features of up-regulated DEGs. (**C**) Circos plots show overlapping and specific responses of down-regulated DEGs. (**D**) Circos plots summarize features of down-regulated DEGs.

The 721 up-regulated genes were significantly enriched in 65 KEGG pathways, in which 22 pathways containing 119 up-regulated genes were involved in inflammation and immune response (Table 2). The 381 down-regulated genes are significantly enriched in 26 KEGG pathways, in which 10 KEGG pathways containing 51 down-regulated genes were involved in inflammation and immune response (Table 2). The top 30 up-regulated and down-regulated pathways are listed in Figure 7. Features of DEGs enriched in the top 9 significant KEGG terms are shown in Figure 8.

### 2.7. Validation of DE miRNAs and DEGs by qRT-PCR

To verify the accuracy of RNA sequencing and microarray, qRT-PCR was performed to detect the expression levels of miRNA and DEGs. The results showed that the relative expression levels of selected miRNAs and mRNAs identified by qRT-PCR were consistent with RNA sequencing and microarray results, respectively (Tables S4 and S5), indicating a high reliability of the study.

## 3. Discussion

**Figure 9:**
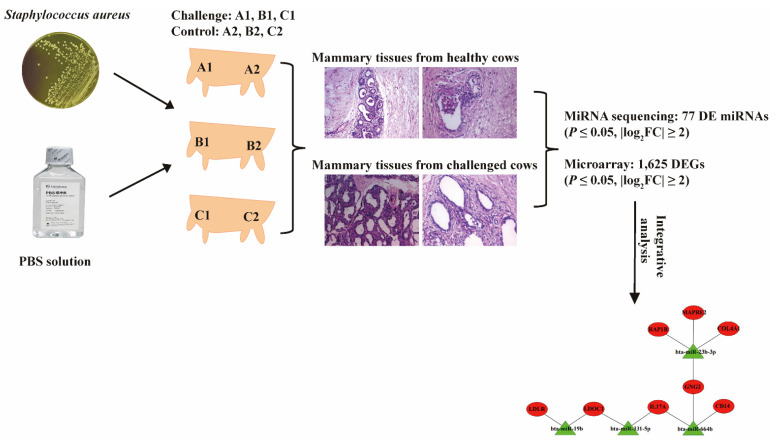
The construction of *Staphylococcus aureus*-induced mastitis and pathological features and integrative analysis of miRNA and mRNA expression profiles of mammary tissues.

To date, more than 150 pathogenic bacteria have been identified in dairy cows with mastitis; among them, *Escherichia coli*, *Streptococcus* spp. and *S. aureus* are most frequently isolated from cows with clinical or subclinical mastitis [9,32]. In this study, the *S. aureus*-type bovine mastitis model was constructed to explore interaction patterns of mRNAs and miRNAs in the *S. aureus*-infected quarters and the control ones. One quarter of the mammary gland of each cow received the inoculation of *S. aureus*, and the remaining quarters with the inoculation of PBS served as control group. In this way, the systematic errors could be well minimized when we analyzed and compared the expression levels of mRNAs and miRNAs between inoculated and control groups [33,34]. In total, 77 DE miRNAs and 1625 DEGs were identified in the *S. aureus*-challenged quarters, compared with the healthy ones (Figure 9).

A previous study showed that miR-664b is a promising candidate involved in response to pathogen infection, which was down-regulated in *S. aureus*-infected quarters (0.450-fold change, *p* < 0.001) [35]. Accordingly, *CD14 Molecule* (*CD14*), a lipopolysaccharide-binding protein enriched significantly in several inflammation-related terms (cellular response to organic substance/oxygen-containing compound/biotic stimulus/biotic stimulus/molecule of bacterial origin terms), which was identified as a predicted target of miR-664b, was up-regulated in *S. aureus*-infected quarters (2.151-fold change, *p* = 0.002) (Table S6). This result is consistent with previous studies, in which *CD14* was measured as an up-regulated trend as an early innate immune response gene in bacterial infections of mammary gland [13,36,37]. This finding potentially supports that miR-664b negatively regulates its target gene, *CD14*, to mediate inflammation in mammary gland of dairy cattle infected by *S. aureus*.

*G protein subunit gamma 2* (*GNG2*), another target gene of miR-664b, was up-regulated in *S. aureus*-inoculated quarters (3.246-fold change, *p* = 0.020), which is significantly enriched in three significant terms (cellular response to organic substance term, cellular response to oxygen-containing compound term and cellular response to acid chemical term) and four significant pathways (PI3K–Akt signaling pathway, chemokine signaling pathway, Kaposi sarcoma-associated herpesvirus infection pathway and Ras signaling pathway) (Table S6). These terms and pathways are mainly involved in inflammation response. Previous studies mainly focused on functional analysis of *GNG2* in human malignant melanoma cells [38,39,40]. However, there is no direct evidence to prove the association between the up-regulation of *GNG2* and the infection of *S. aureus* in mammary glands. The highly expressed *GNG2* may also be associated with the down-regulation of miR-23b-3p (0.223-fold change, *p* < 0.001), which was identified to be associated with various cancers, such as cervical cancer, renal cancer and pancreatic cancer [41,42,43,44]. Other up-regulated DEGs regulated by miR-23b-3p in the *S. aureus* infection group were *collagen type IV alpha 1 chain* (*COL4A1*) (2.272-fold change, *p* = 0.007), *microtubule associated protein RP/EB family member 2* (*MAPRE2*) (5.500-fold change, *p* = 0.001) and *member of RAS oncogene family* (*RAP1B*) (2.548-fold change, *p* = 0.008). Although *COL4A1*, *MAPRE2* and *RAP1B* are respectively enriched in various inflammation-related terms and pathways, to our knowledge, there is no evidence to prove that they have a bearing on bovine mastitis infected by *S. aureus*.

The down-regulation of miR-664b has a potential association with the extremely significant up-regulation of *interleukin 17A* (*IL17A*) (18.584-fold change, *p* < 0.001) in *S. aureus*-inoculated quarters, which plays a crucial role in the defense of Gram-positive bacterial infection and inflammation development [45,46,47]. *IL17A* is significantly enriched in the terms of cellular response to organic substance, leukocyte migration and inflammatory response and the pathways of IL-17 signaling and rheumatoid arthritis, which indicated that *IL17A* potentially acts as a functional gene in the defense of *S. aureus* infection in bovine mammary glands. Generally known, the expression level of a single gene can be regulated by multiple miRNAs [48]. As shown in this study, miR-331-5p, which targets *IL17A*, was down-regulated in *S. aureus*-inoculated quarters (0.273-fold change, *p* < 0.001). At the same time, *LDOC1 regulator of NFKB signaling* (*LDOC1*), the target gene of miR-331-5p, was up-regulated in the infected group (2.114-fold change, *p* = 0.002). *LDOC1* is significantly enriched in cellular response to organic substance term, cellular response to oxygen-containing compound term, cellular response to biotic stimulus term, cellular response to lipopolysaccharide term, response to lipopolysaccharide term, cellular response to molecule of bacterial origin term and response to molecule of bacterial origin term. Previous studies have suggested that LDOC1 regulated the expression of *nuclear factor kappa-B* (*NF-κB*), which plays a significant role in cellular inflammatory and immune responses [49]. Additionally, multiple studies have shown that LDOC1 can induce apoptosis [50,51,52]. Thus, it remains to be clarified the role of LDOC1 in *S. aureus*-induced apoptosis.

The down-regulation of miR-19b (0.397-fold change, *p* < 0.001) is potentially responsible for the up-regulation of LDOC1 in *S. aureus*-induced mastitis, which has been identified to be the candidate marker for lung cancer and diabetes [53,54]. The down-regulation of miR-19b is also observed to account for the down-regulation of *low-density lipoprotein receptor* (*LDLR*) (2.976-fold change, *p* = 0.024), which was significantly enriched in cellular response to organic substance term, cellular response to oxygen-containing compound term, cellular response to acid chemical term, inflammatory response term and toxoplasmosis pathway and can develop inflammatory atherosclerosis [55].

*S100 calcium binding protein A9* (*S100A9*) is a kind of pro-inflammatory factor, and the protein from exosomes in follicular fluid causes inflammation by NF-κB pathway activation in polycystic ovary syndrome [56,57]. In this study, the up-regulated *S100A9* (10.631-fold change, *p* = 0.006) and down-regulated predicted target miRNA-2431-3p (0.459-fold change, *p* = 0.005) were screened in *S. aureus*-inoculated quarters. *S100A9* was enriched in multiple significant inflammatory and immune-related pathways, including positive regulation of hydrolase activity pathway, leukocyte migration pathway, neutrophil chemotaxis pathway and inflammatory response pathway.

## 4. Materials and Methods

### 4.1. Ethics Statement and Animals Selection

All experimental protocols in this study were reviewed and approved by the Institutional Animal Care and Use Committee of Yangzhou University (ZZCX2019-SYXY-056). All methods in this study were carried out in accordance with the Administration of Affairs Concerning Experimental Animals published by the Ministry of Science and Technology of China.

Three apparently half-sib, healthy and mastitis-free Holstein dairy cattle (A, B and C) were chosen from a dairy farm in Yangzhou, China. All the three cows were in the middle lactation term of first parity with a consistent history of milk somatic cell count (SCC) below 100,000/mL. In particular, the employed cows were detected to be in absence of *Mycobacterium bovis*, *Brucella abortus*, *Anaplasma* spp., *Babesia* spp., *Theileria* spp., bovine leukemia virus, bovine herpesvirus-1, bovine viral diarrhea virus and bovine respiratory syncytial virus with commercial or in-house molecular diagnostic kits [58,59,60,61]. Then, the experiment was performed after one week in quarantine.

### 4.2. Mastitis Model Construction

For challenge infection study, aliquots from frozen stock cultures (*S. aureus*, ATCC29213) were plated on sheep blood agar and incubated at 37 °C for 18 h under 10% CO2-enriched conditions. Bacterial suspensions for each pure culture were diluted in sterile phosphate buffered saline (PBS) (Biosharp, Hefei, China) to 1 × 10^7^ Colony-Forming Units (CFU)/mL, using a spectrophotometer (Eppendorf, Germany) with a wavelength of 600 nm. For challenged group, one quarter (A1, B1 and C1) of the mammary gland of the three individuals received a dose of 5 × 10^7^ CFU of *S. aureus*, and one of the remaining quarters (A2, B2 and C2) not administered with the *S. aureus* inoculation served as control group that received 5 mL of sterile PBS [20,62]. The milk yield, SCC (Shanghai DHI Test Center, Shanghai, China) and temperature of cows were recorded before and at 24 h post-inoculation.

### 4.3. Sample Collection and Total RNA Extraction

The mammary tissues (1–2 g per quarter) were collected by sterile surgery from two quarters per dairy cattle at 48 h post-inoculation. Samples from challenged (A1, B1 and C1) and control (A2, B2 and C2) quarters were immediately frozen in liquid nitrogen before RNA extraction or stored in 10% formalin for hematoxylin and eosin (HE) staining.

Total RNA was extracted from 250 mg mammary tissues with mirVanaTM RNA Isolation Kit (Applied Biosystems, Carlsbad, CA, USA) and purified with QIAGEN RNeasy^®^ Kit (QIAGEN, Dusseldorf, Germany). The RNA quality was assessed using Agilent Bioanalyzer 2100 (Agilent Technologies, Santa Clara, USA) and NanoDrop spectrophotometer (Thermo Fisher, USA). Total RNA samples were stored at −70 °C. A total of 10 μg per RNA sample was sent to a commercial sequencing laboratory (Oebiotech, Shanghai, China) for evaluating the expression levels of miRNA with HiSeq 2000 System (single-end) (Illumina, San Diego, CA, USA) and mRNA with microarray (G2519F-023647, Agilent Technologies, Santa Clara, CA, USA).

### 4.4. Pathological Tests

After 48 h of soaking, the samples were rinsed with water for 12 h and subjected to gradient alcohol dehydration, wax impregnation and embedding. Hematoxylin-eosin (HE) staining was performed for 15 min after dewaxing and adequate washing. The pathological changes were visualized with a microscope (M152, Mshot, Guangzhou, China) at different magnifications.

### 4.5. Small RNA Sequencing and Data Analysis

Clean reads constructing the small RNA libraries were obtained by removing low-quality reads, adaptors and insufficient tags. Then the length distribution and sequences of the clean reads were summarized and analyzed, respectively. Ribosomal RNAs (rRNAs), transfer RNAs (tRNAs) and other noncoding RNAs were identified and removed, based on GenBank (http://www.ncbi.nlm.nih.gov, accessed on 6 October 2020) and Rfamdatabase10.1 (http://rfam.xfam.org/, accessed on 6 October 2020). MiRNAs were identified through a BLASTN search against the miRBase18.0 (http://www.mirbase.org/, accessed on 6 October 2020) [63].

The miRNA counts were normalized as transcript per million (TPM) with the formula (number of reads per miRNA alignment) / (number of reads from the total sample alignment) × 10^6^ [64]. The differentially expressed (DE) miRNAs in each sample were calculated with DEseq R package (1.18.0), with *p* ≤ 0.05 and fold change ≥2 as the threshold.

### 4.6. mRNA Analysis and Data Process

The 2100 RNA Integrity Number (RIN) and 28S/18S values were detected to evaluate the quality of RNAs. The GeneSpring software (version 12.5, Agilent Technologies, Santa Clara, CA, USA) was utilized to evaluate the coefficient of variation (CV) of each sample.

Total RNA was reverse-transcribed to double-stranded complementary DNA (cDNA) and purified with QIAGEN RNeasy^®^ Kit (QIAGEN, Dusseldorf, Germany), from which cNDAs were synthesized and then labeled with cyanine-3-cytidine triphosphate. For the calculation of fluorescence molecule concentration and incorporation, the following formulas were employed: Cy3-concentration (pmol/µL) = A552/0.15, and Cy3-incorproation (pmol/µg) = Cy3-concentration/cRNA concentration (µg/µL). Then, the cDNA sample fragmentation and chip hybridization were conducted, and the chips were washed and scanned subsequently.

Feature Extraction software (version 10.7.1.1, Agilent Technologies Santa Clara, CA, USA) was employed to extract and analyze raw data from array images. Briefly, the raw data was normalized with the quantile algorithm, and the resultant flag value of any probe was assigned as “Detected” only if there were no “Compromised” or “Not Detected”. DEGs were identified with *p* ≤ 0.05 and |log_2_FC| ≥ 1 as the threshold.

### 4.7. miRNA–mRNA Interaction Network Construction

With the online software TargetScan (www.targetscan.org, accessed on 6 November 2020), the potential target genes of DE miRNAs with more significant expression levels (*p* ≤ 0.05 and |log_2_FC| ≥ 2) were predicted and intersected, with the DEGs identified by microarray test (*p* ≤ 0.05 and |log_2_FC| ≥ 2). Then, the miRNA–mRNA interaction networks were constructed and visualized with the DE miRNAs and screened genes by Cytoscape (v3.7.2) [65].

To evaluate the reliability of the miRNA–mRNA interaction network, the DE miRNAs and DEGs (*p* ≤ 0.05 and |log_2_FC| ≥ 2) obtained in this study were compared and taken the intersections with those from previous relevant studies [20,22,23,24,25,26,27,28,29,30,31].

### 4.8. Functional Analysis of Differentially Expressed Genes

DEGs regulated by DE miRNAs were screened to further understand their biological and metabolic pathways. Gene ontology (GO) annotation and Kyoto Encyclopedia of Genes and Genomes (KEGG) analysis were respectively performed with the DAVID 6.8 (https://david.ncifcrf.gov/, accessed on 6 November 2020) and KOBAS 3.0 (http://kobas.cbi.pku.edu.cn/index.php, accessed on 6 November 2020) using R based on the hypergeometric distribution [65]. Then, the GO terms and KEGG pathways with adjusted *p* ≤ 0.05 were significantly enriched in DEGs or the miRNA target genes.

### 4.9. RT-qPCR Validation of DEGs and DE miRNAs

To validate the RNA sequencing data, five duplicates of eight DEGs (*DGAT2*, *FADS2*, *ALDH3A2*, *EHHADH*, *FASN*, *LPL*, *SCD* and *SLC27A6*) and six DE miRNAs (bta-miR-196a, bta-miR-205, bta-miR-200b, bta-miR-223, bta-miR-184, bta-miR-1246) were selected and analyzed by RT-qPCR. All the specific primers were synthesized by a commercial company (Sangon Biotech, Shanghai, China) and are described in Tables S7 and S8. The LightCycler^®^ 480 II System (Roche, Basel, Switzerland) was applied to qRT-PCR with 20 μL volumes composed of 10 μL of 2 × TB Green Fast qPCR Mix (Takara, Dalian, China), 0.8 μL of forward/ reverse primer, 2 μL of DNA template and 6.4 μL of double distilled water (ddH_2_O). Thermal cycling consisted of a 30 s denaturation step at 94 °C, followed by 40 cycles of 94 °C for 5 s and 60 °C for 30 s, melting curve determination between 50 °C and 90 °C and final holding at 37 ℃. MiRNA/mRNA were normalized for bovine *18S rRNA*/*β-actin*. Relative expression was calculated using the 2^−ΔΔCt^ method in all samples.

### 4.10. Statistical Analysis

Data were analyzed using GraphPad Prism 8 (GraphPad, San Diego, CA, USA) with Student’s *t*-test and presented as mean ± standard deviation (SD). The resulting *p*-values were adjusted using the Benjamini and Hochberg’s approach for controlling the false discovery rate (FDR). Adjusted *p* < 0.05 indicated a significant difference.

## 5. Conclusions

In the present study, we comprehensively analyzed the changes in miRNA and mRNA profiles of the mammary gland of dairy cattle under *S. aureus* inoculation. Overall, 77 DE miRNAs and 1625 DEGs were identified in the *S. aureus*-challenged quarters. Among them, the predicted integrated regulatory network was constructed with the miRNAs (miR-664b, miR-23b-3p, miR-331-5p, miR-19b and miR-2431-3p) and the mRNAs (*CD14*, *GNG2*, *COL4A1*, *MAPRE2*, *RAP1B*, *IL17A*, *LDOC1*, *LDLR* and *S100A9*), which were significantly associated with inflammation and immunity. These findings could enhance the understanding of underlying immune response in bovine mammary glands against *S. aureus* infection and provide a useful foundation for the future application of the miRNA–mRNA-based genetic regulatory network in the breeding of cows resistant to *S. aureus*.

## Figures and Tables

**Table 1:** Significant terms involved in inflammation and immune response.

Term ID	Term	*P*-Value	Gene Name	Number
GO:0071310	cellular response to organic substance	0.009	***CXCR1***^a^, ***GFPT2***, ***CSF3***, ***IL17A***, ***PTGS2***, ***WNT2***, ***CXCL5***, ***IL2RA***, ***OAS2***, ***PTAFR***, ***PTGIS***, ***ABHD2***, ***RIPOR2***, ***SOCS3***, ***COL1A2***, ***GNG2***, ***COL1A1***, ***SCARB1***, ***LDLR***, ***FYN***, ***ATP2B4***, ***SNAI2***, ***MSN***, ***IRAK2***, ***RAP1B***, ***WASF1***, ***CD14***, ***COL4A1***, ***DERL1***, ***HSPA5***, ***ACVR2A***, ***LDOC1***, ***EHD1***, ***UFM1***	34
GO:0051345	positive regulation of hydrolase activity	0.010	***SELE***, ***S100A9***, ***HTR2A***, ***MAPRE2***, ***AHSA2***, ***PYCARD***, ***ABR***, ***CHN1***, ***DNAJB4***, ***ARHGAP15***, ***SEC23A***, ***ATP1B3***, ***AGFG1***, ***ASAP1***	14
GO:1901701	cellular response to oxygen-containing compound	0.011	***CXCL5***, ***PTAFR***, ***COL1A2***, ***GNG2***, ***COL1A1***, ***SCARB1***, ***LDLR***, ***FYN***, ***ATP2B4***, ***TXN***, ***SNAI2***, ***MSN***, ***IRAK2***, ***RAP1B***, ***CD14***, ***COL4A1***, ***LDOC1***, ***NCF1***, ***SOD2***	19
GO:0071216	cellular response to biotic stimulus	0.015	***CXCL5***, ***PTAFR***, ***SCARB1***, ***IRAK2***, ***CD14***, ***HSPA5***, ***LDOC1***	7
GO:0071222	cellular response to lipopolysaccharide	0.015	***CXCL5***, ***PTAFR***, ***SCARB1***, ***IRAK2***, ***CD14***, ***LDOC1***	6
GO:0072676	lymphocyte migration	0.016	***RIPOR2***, ***PYCARD***, ***STK10***, ***MSN***	4
GO:0032496	response to lipopolysaccharide	0.020	***CXCL5***, ***PTAFR***, ***SCARB1***, ***IRAK2***, ***TBXA2R***, ***CD14***, ***LDOC1***	7
GO:0071219	cellular response to molecule of bacterial origin	0.021	***CXCL5***, ***PTAFR***, ***SCARB1***, ***IRAK2***, ***CD14***, ***LDOC1***	6
GO:0030334	regulation of cell migration	0.023	***SRPX2***, ***PRR5L***, ***ABHD2***, ***RIPOR2***, ***STC1***, ***MAPRE2***, ***MMP14***, ***PYCARD***, ***COL1A1***, ***STK10***, ***SNAI2***, ***MSN***, ***TBXA2R***, ***ITGB1***, ***HSPA5***	15
GO:0071229	cellular response to acid chemical	0.028	***COL1A2***, ***GNG2***, ***COL1A1***, ***LDLR***, ***COL4A1***	5
GO:0032729	positive regulation of interferon gamma production	0.028	***PYCARD***, ***FAM49B***, ***CD14***	3
GO:0050900	leukocyte migration	0.030	***SELE***, ***IL17A***, ***S100A9***, ***CXCL5***, ***RIPOR2***, ***PYCARD***, ***STK10***, ***MSN***	8
GO:0002237	response to molecule of bacterial origin	0.031	***CXCL5***, ***PTAFR***, ***SCARB1***, ***IRAK2***, ***TBXA2R***, ***CD14***, ***LDOC1***	7
GO:0030593	neutrophil chemotaxis	0.034	***S100A9***, ***CXCL5***, ***RIPOR2***	3
GO:0072678	T cell migration	0.034	***RIPOR2***, ***PYCARD***, ***MSN***	3
GO:0006954	inflammatory response	0.038	***IL17A***, ***S100A9***, ***THBS1***, ***PTGS2***, ***ALOX5AP***, ***CD163***, ***PTGS1***, ***PTAFR***, ***PTGIS***, ***SOCS3***, ***PYCARD***, ***LDLR***, ***IRAK2***, ***CYBB***	14
GO:0030203	glycosaminoglycan metabolic process	0.040	***LYVE1***, ***DSE***, ***SLC35D1***, ***UGDH***	4
GO:0050954	sensory perception of mechanical stimulus	0.040	***RIPOR2***, ***COL1A1***, ***FYN***, ***SNAI2***	4
GO:0071230	cellular response to amino acid stimulus	0.041	***COL1A2***, ***COL1A1***, ***COL4A1***	3
GO:0006935	chemotaxis	<0.001	*CXCL11*^b^, *CXCL10*, *F2RL1*, *CXCL9*, *MSTN*, *NFIB*, *MET*, *CCL5*, *PDGFA*, *CXCR3*, *SCN1B*	11
GO:0050921	positive regulation of chemotaxis	<0.001	*CXCL10*, *F2RL1*, *MSTN*, *MET*, *CCL5*, *CXCR3*	6
GO:0050920	regulation of chemotaxis	<0.001	*CXCL10*, *F2RL1*, *MSTN*, *MET*, *CCL5*, *CXCR3*	6
GO:0032103	positive regulation of response to external stimulus	0.001	*CXCL10*, *F2RL1*, *MSTN*, *C3*, *MET*, *CCL5*, *CXCR3*	7
GO:0050900	leukocyte migration	0.001	*CXCL11*, *CXCL10*, *F2RL1*, *MSTN*, *GATA3*, *CCL5*, *CXCR3*	7
GO:0060326	cell chemotaxis	0.004	*CXCL11*, *CXCL10*, *MSTN*, *MET*, *CCL5*	5
GO:0002690	positive regulation of leukocyte chemotaxis	0.005	*CXCL10*, *MSTN*, *CCL5*	3
GO:1990868	response to chemokine	0.005	*CX3CR1*, *CCL5*, *CXCR3*	3
GO:1990869	cellular response to chemokine	0.005	*CX3CR1*, *CCL5*, *CXCR3*	3
GO:0032101	regulation of response to external stimulus	0.006	*CXCL10*, *F2RL1*, *MSTN*, *S100B*, *C3*, *GATA3*, *MET*, *CCL5*, *PDGFA*, *CXCR3*	10
GO:0002688	regulation of leukocyte chemotaxis	0.010	*CXCL10*, *MSTN*, *CCL5*	3
GO:0002685	regulation of leukocyte migration	0.012	*CXCL10*, *MSTN*, *CCL5*, *CXCR3*	4
GO:0030595	leukocyte chemotaxis	0.013	*CXCL11*, *CXCL10*, *MSTN*, *CCL5*	4
GO:0002687	positive regulation of leukocyte migration	0.016	*CXCL10*, *MSTN*, *CCL5*	3
GO:0007606	sensory perception of chemical stimulus	0.027	*SCNN1G*, *SCNN1B*	2
GO:0036230	granulocyte activation	0.027	*F2RL1*, *CCL5*	2
GO:0071622	regulation of granulocyte chemotaxis	0.027	*MSTN*, *CCL5*	2
GO:1905517	macrophage migration	0.027	*MSTN*, *CCL5*	2
GO:0002673	regulation of acute inflammatory response	0.032	*S100B*, *C3*	2
GO:0050918	positive chemotaxis	0.034	*CXCL10*, *MET*, *CCL5*	3
GO:0009605	response to external stimulus	0.039	*CXCL11*, *CXCL10*, *F2RL1*, *CXCL9*, *MSTN*, *S100B*, *C3*, *NFIB*, *REEP6*, *GATA3*, *AQP3*, *MET*, *IKZF3*, *CCL5*, *PDGFA*, *CXCR3*, *SCN1B*	17
GO:0072678	T cell migration	0.043	*CXCL11*, *CXCL10*	2
GO:2000401	regulation of lymphocyte migration	0.048	*CXCL10*, *CCL5*	2
GO:1904062	regulation of cation transmembrane transport	0.048	*CXCL11*, *CXCL10*, *CXCL9*, *CXCR3*	4
GO:0042379	chemokine receptor binding	<0.001	*CXCL11*, *CXCL10*, *CXCL9*, *CCL5*	4

^a^ The names in bold italic indicate that the genes are up-regulated in the *S. aureus*-inoculated group. ^b^ The names in regular italic indicate that the genes are down-regulated in the *S. aureus*-inoculated group.

**Table 2:** Significant KEGG pathways involved in inflammation and immune response.

Pathway ID	Pathway	*P*-Value	Gene Name	Number
bta04666	Fc gamma R-mediated phagocytosis	<0.001	***PLA2G4A*^a^**, ***MARCKSL1***, ***VASP***, ***SYK***, ***PIK3R3***, ***FCGR1A***, ***WASF1***, ***CFL1***, ***ASAP1***, ***NCF1***, ***ARPC5***, ***LYN***, ***ARPC2***, ***MAP2K1***	14
bta04668	TNF signaling pathway	<0.001	***CXCL2***, ***SELE***, ***MMP3***, ***PTGS2***, ***CXCL6***, ***VEGFC***, ***SOCS3***, ***MMP14***, ***CASP3***, ***LIF***, ***CSF1***, ***PIK3R3***, ***MAP3K8***, ***TNFAIP3***, ***MAP2K3***, ***MAP2K1***	16
bta04066	HIF-1 signaling pathway	<0.001	***SERPINE1***, ***LDHA***, ***PFKFB3***, ***PGK1***, ***HIF1A***, ***PFKP***, ***TFRC***, ***PIK3R3***, ***MKNK1***, ***ALDOA***, ***ENO1***, ***ENO2***, ***CYBB***, ***MAP2K1***	14
bta04015	Rap1 signaling pathway	<0.001	***ITGAM***, ***THBS1***, ***PDGFRA***, ***ID1***, ***ITGB3***, ***PDGFD***, ***VEGFC***, ***APBB1IP***, ***FYB***, ***PDGFRB***, ***VASP***, ***CSF1***, ***PIK3R3***, ***SIPA1L2***, ***RAP1B***, ***PFN1***, ***MAP2K3***, ***ITGB1***, ***TLN1***, ***PRKD3***, ***MAP2K1***	21
bta04657	IL-17 signaling pathway	<0.001	***CXCL2***, ***CSF3***, ***IL17A***, ***MMP3***, ***S100A9***, ***FOSL1***, ***PTGS2***, ***CXCL6***, ***MMP1***, ***CASP3***, ***TNFAIP3***, ***MAPK6***	12
bta05020	Prion diseases	0.001	***NCAM1***, ***LAMC1***, ***FYN***, ***PRKACB***, ***HSPA5***, ***MAP2K1***	6
bta04664	Fc epsilon RI signaling pathway	0.002	***ALOX5AP***, ***FCER1A***, ***PLA2G4A***, ***FYN***, ***SYK***, ***PIK3R3***, ***MAP2K3***, ***LYN***, ***MAP2K1***	9
bta04151	PI3K–Akt signaling pathway	0.002	***CSF3***, ***THBS2***, ***BDNF***, ***THBS1***, ***ITGA5***, ***IL2RA***, ***PDGFRA***, ***EPOR***, ***ITGB3***, ***PDGFD***, ***VEGFC***, ***COL1A2***, ***LAMA4***, ***ITGA9***, ***LAMC1***, ***GNG2***, ***COL1A1***, ***PDGFRB***, ***CSF1***, ***SYK***, ***PIK3R3***, ***YWHAG***, ***GNB4***, ***COL4A1***, ***ITGB1***, ***CDK2***, ***MAP2K1***	27
bta05134	Legionellosis	0.002	***CXCL2***, ***ITGAM***, ***NAIP***, ***CASP3***, ***PYCARD***, ***HSPA8***, ***CD14***, ***SAR1A***	8
bta05146	Amoebiasis	0.002	***SERPINB4***, ***CXCL2***, ***ITGAM***, ***COL1A2***, ***CASP3***, ***LAMA4***, ***LAMC1***, ***COL1A1***, ***PRKACB***, ***PIK3R3***, ***CD14***, ***COL4A1***	12
bta04670	Leukocyte transendothelial migration	0.005	***ITGAM***, ***MMP2***, ***JAM3***, ***VASP***, ***PIK3R3***, ***MSN***, ***RAP1B***, ***PTPN11***, ***ITGB1***, ***NCF1***, ***CYBB***	11
bta04062	Chemokine signaling pathway	0.007	***CXCR2***, ***CXCL2***, ***CCR1***, ***CXCL6***, ***CCL16***, ***PREX1***, ***GNG2***, ***ARRB2***, ***PRKACB***, ***PIK3R3***, ***RAP1B***, ***GNB4***, ***NCF1***, ***LYN***, ***MAP2K1***	15
bta05100	Bacterial invasion of epithelial cells	0.008	***ITGA5***, ***CBL***, ***PIK3R3***, ***WASF1***, ***DNM3***, ***ITGB1***, ***ARPC5***, ***ARPC2***	8
bta04145	Phagosome	0.008	***THBS2***, ***ITGAM***, ***THBS1***, ***ITGA5***, ***ITGB3***, ***SCARB1***, ***TUBB3***, ***TFRC***, ***FCGR1A***, ***CD14***, ***ITGB1***, ***ATP6V1C1***, ***NCF1***, ***CYBB***	14
bta05165	Human papillomavirus infection	0.011	***THBS2***, ***THBS1***, ***PTGS2***, ***WNT2***, ***ITGA5***, ***ITGB3***, ***PKM***, ***COL1A2***, ***CASP3***, ***LAMA4***, ***ITGA9***, ***LAMC1***, ***COL1A1***, ***PDGFRB***, ***PRKACB***, ***NOTCH2***, ***PIK3R3***, ***COL4A1***, ***MX2***, ***ITGB1***, ***ATP6V1C1***, ***CDK2***, ***MAP2K1***	23
bta05167	Kaposi sarcoma-associated herpesvirus infection	0.023	***CXCL2***, ***CCR1***, ***PTGS2***, ***E2F3***, ***CASP3***, ***PREX1***, ***GNG2***, ***HIF1A***, ***RCAN1***, ***SYK***, ***PIK3R3***, ***GNB4***, ***MAPKAPK2***, ***LYN***, ***MAP2K1***	15
bta05323	Rheumatoid arthritis	0.023	***CXCL2***, ***IL17A***, ***MMP3***, ***CXCL6***, ***MMP1***, ***CD80***, ***CSF1***, ***ATP6V1C1***, ***IL11***	9
bta04392	Hippo signaling pathway- multiple species	0.026	***RASSF2***, ***WTIP***, ***TEAD3***, ***WWTR1***	4
bta04014	Ras signaling pathway	0.030	***BDNF***, ***PDGFRA***, ***PDGFD***, ***VEGFC***, ***PLA2G4A***, ***GNG2***, ***PDGFRB***, ***PRKACB***, ***CSF1***, ***PIK3R3***, ***RAP1B***, ***GNB4***, ***ABL1***, ***PTPN11***, ***ABL2***, ***MAP2K1***	16
bta04061	Viral protein interaction with cytokine and cytokine receptor	0.033	***CXCR2***, ***CXCL2***, ***CCR1***, ***CXCL6***, ***IL2RA***, ***CCL16***, ***IL10RA***, ***CSF1***	8
bta05140	Leishmaniasis	0.033	***ITGAM***, ***PTGS2***, ***MARCKSL1***, ***FCGR1A***, ***ITGB1***, ***NCF1***, ***CYBB***	7
bta05145	Toxoplasmosis	0.035	***IL10RA***, ***CASP3***, ***LAMA4***, ***LAMC1***, ***LDLR***, ***SOCS1***, ***MAP2K3***, ***HSPA8***, ***ITGB1***	9
bta04060	Cytokine–cytokine receptor interaction	<0.001	*CXCL11*^b^, *CX3CR1*, *CXCL10*, *CXCL9*, *NGFR*, *CXCL14*, *MSTN*, *XCL1*, *IL17RE*, *BMP3*, *TNFRSF9*, *TNFSF10*, *GHR*, *CXCR6*, *CCL5*, *TNFRSF19*, *CXCR3*, *TGFB2*	18
bta04061	Viral protein interaction with cytokine and cytokine receptor	<0.001	*CXCL11*, *CX3CR1*, *CXCL10*, *CXCL9*, *CXCL14*, *XCL1*, *TNFSF10*, *CCL5*, *CXCR3*	9
bta04062	Chemokine signaling pathway	0.001	*CXCL11*, *CX3CR1*, *CXCL10*, *CXCL9*, *CXCL14*, *XCL1*, *ITK*, *PRKCZ*, *CXCR6*, *CCL5*, *CXCR3*	11
bta04015	Rap1 signaling pathway	0.004	*FGFR4*, *NGFR*, *PRKCZ*, *FGFR2*, *MET*, *LPAR2*, *TLN2*, *INSR*, *PDGFA*, *MAGI3*, *CTNND1*	11
bta04670	Leukocyte trans endothelial migration	0.007	*CLDN1*, *ITK*, *OCLN*, *CLDN3*, *TXK*, *EZR*, *CTNND1*	7
bta05340	Primary immunodeficiency	0.009	*CD8A*, *BLNK*, *CIITA*, *TAP1*	4
bta01521	EGFR tyrosine kinase inhibitor resistance	0.020	*ERBB3*, *FGFR2*, *MET*, *PDGFA*, *GAB1*	5
bta04010	MAPK signaling pathway	0.028	*FGFR4*, *ERBB3*, *NGFR*, *RPS6KA6*, *FGFR2*, *MET*, *INSR*, *MAP3K13*, *PDGFA*, *MAP3K1*, *TGFB2*	11
bta04390	Hippo signaling pathway	0.034	*RASSF6*, *PRKCZ*, *DLG3*, *PPP2R2B*, *TCF7*, *TCF7L2*, *TGFB2*	7
bta04151	PI3K–Akt signaling pathway	0.035	*FGFR4*, *ERBB3*, *NGFR*, *GHR*, *FGFR2*, *PPP2R2B*, *MET*, *LPAR2*, *INSR*, *ITGA6*, *LAMC2*, *PDGFA*, *ITGA3*	13

^a^ The names in bold italic indicate that the genes are up-regulated in the *S. aureus*-inoculated group. ^b^ The names in regular italic indicate that the genes are down-regulated in the *S. aureus*-inoculated group.

## Data Availability

The data presented in this study are available in the main text and supplementary material of this article.

## Supplementary Materials

The following are available online at https://www.mdpi.com/article/10.3390/pathogens10050506/s1. Table S1: Statistics of miRNA sequencing. Table S2: The quality control of mRNAs. Table S3: The variation coefficient of samples used for microarray test. Table S4: Comparison of the expression levels of seven miRNAs detected by transcriptome sequencing and qRT-PCR. Table S5: Comparison of the expression levels of eight mRNAs detected by microarray and qRT-PCR. Table S6: Functional annotations of key DEGs and their potential target miRNAs. Table S7: The primers used for qRT-PCR to validate the small RNA sequencing. Table S8: The primers used for qRT-PCR to validate the microarray test. Figure S1: The length distribution of small RNAs in (A) control group and (B) *S. aureus*-inoculated group.
