# Supplementary material for: Integrative Analysis of miRNA and mRNA Expression Profiles in Mammary Glands of Holstein Cows Artificially Infected with Staphylococcus aureus
Source: Pathogens. 2021 Apr 22;10(5):506. doi: 10.3390/pathogens10050506 (PMC8145100; doi:10.3390/pathogens10050506)
Supplement: Supplementary file 1 [file pathogens-10-00506-s001.zip › pathogens-1179550-supplementary.pdf]

Table S1. Statistics of miRNA sequencing.

Type	Control Group		*S. aureus* Group	
	Count	Percent (%)	Count	Percent (%)
Total reads	21,293,853		18,588,177	
high_quality	21,253,265	100	18,565,803	100
3' adapter_null	5959	0.03	3756	0.02
insert_null	8499	0.04	3612	0.02
5' adapter_contaminants	330,968	1.56	22,295	0.12
smaller_than_18nt	60,374	0.28	31,238	0.17
polyA	465	0.00	127	0.00
clean_reads	20,847,000	98.09	18,504,775	99.67

Table S2. The quality control of mRNAs.

Sample	A260/A280	Volume (μL)	28S/18S	RIN
A1	2.10	95	1.3	7.9
B1	2.11	95	1.6	8.7
C1	2.06	95	1.5	7.5
A2	2.10	95	2.1	8.5
B2	2.11	95	1.5	8.9
C2	2.12	95	1.9	8.5

Table S3. The variation coefficient of samples used for microarray test.

Sample	Chip number	Fluorescent mark	CV (%)	Result
A1	10691-1	cy3	3.38921	Success
B1	10692-1	cy3	4.26303	Success
C1	10693-1	cy3	3.66861	Success
A2	10691-2	cy3	4.82096	Success
B2	10692-3	cy3	4.03989	Success
C2	10693-4	cy3	3.78828	Success

Table S4. Comparison of the expression levels of seven miRNAs detected by transcriptome sequencing and qRT-PCR.

miRNAs	miRNA-seq Log$_2$(fold change)	qRT-PCR Log$_2$(fold change)
bta-miR-196a	-1.14	-1.53
bta-miR-205	-1.36	-1.07
bta-miR-200b	-1.05	-1.35
bta-miR-223	+4.88	+5.68
bta-miR-184	+4.82	+4.19
bta-miR-1246	+8.41	+7.56

Table S5. Comparison of the expression levels of eight mRNAs detected by microarray and qRT-PCR.

Genes	Microarray	qRT-PCR
	Log_2(fold change)	Log_2(fold change)
DGAT2	+3.63	+3.85
FADS2	+2.07	+2.21
ALDH3A2	-1.55	-2.04
EHHADH	-2.68	-3.01
FASN	-2.19	-2.12
LPL	-3.42	-2.99
SCD	-1.59	-1.35
SLC27A6	-5.02	-5.26

Table S6. Functional annotations of key DEGs and their potential target miRNAs.

Genes	Log_2(fold change)	Target miRNAs	Go terms	Pathways
CD14	1.25	bta-miR-664b	cellular response to organic substance<br>cellular response to oxygen-containing compound<br>cellular response to biotic stimulus<br>cellular response to lipopolysaccharide<br>response to lipopolysaccharide<br>cellular response to molecule of bacterial origin	none
GNG2	1.84	bta-miR-23b-3p<br>bta-miR-378b<br>bta-miR-664b<br>bta-miR-874	cellular response to organic substance<br>cellular response to oxygen-containing compound<br>cellular response to acid chemical	PI3K-Akt signaling pathway<br>Chemokine signaling pathway<br>Kaposi sarcoma-associated herpesvirus infection<br>Ras signaling pathway
COL4A1	1.20	bta-miR-23b-3p<br>bta-miR-24<br>bta-miR-380-3p	cellular response to organic substance<br>cellular response to oxygen-containing compound<br>cellular response to acid chemical<br>cellular response to amino acid stimulus	PI3K-Akt signaling pathway<br>Amoebiasis<br>Human papillomavirus infection
MAPRE2	2.50	bta-miR-135a<br>bta-miR-19b<br>bta-miR-204<br>bta-miR-23b-3p<br>bta-miR-380-3p<br>bta-miR-410<br>bta-miR-664b	positive regulation of hydrolase activity<br>regulation of cell migration	none
RAP1B	1.35	bta-miR-1<br>bta-miR-139<br>bta-miR-19b<br>bta-miR-200b<br>bta-miR-23b-3p<br>bta-miR-2431-3p<br>bta-miR-26a<br>bta-miR-32<br>bta-miR-380-3p	cellular response to organic substance<br>cellular response to oxygen-containing compound	Rap1 signaling pathway<br>Leukocyte transendothelial migration<br>Chemokine signaling pathway<br>Ras signaling pathway
IL17A	4.22	bta-miR-331-5p<br>bta-miR-664b	cellular response to organic substance<br>leukocyte migration<br>inflammatory response	IL-17 signaling pathway<br>Rheumatoid arthritis
LDOC1	1.12	bta-miR-19b<br>bta-miR-331-5p	cellular response to organic substance<br>cellular response to oxygen-containing compound<br>cellular response to biotic stimulus<br>cellular response to lipopolysaccharide<br>response to lipopolysaccharide<br>cellular response to molecule of bacterial origin<br>response to molecule of bacterial origin	none
LDLR	1.67	bta-miR-19b<br>bta-miR-20a<br>bta-miR-380-3p<br>bta-miR-410<br>bta-miR-450b	cellular response to organic substance<br>cellular response to oxygen-containing compound<br>cellular response to acid chemical<br>inflammatory response	Toxoplasmosis
S100A9	4.07	bta-miR-2431-3p	positive regulation of hydrolase activity<br>leukocyte migration<br>neutrophil chemotaxis<br>inflammatory response	IL-17 signaling pathway

Table S7. The primers used for qRT-PCR to validate the small RNA sequencing.

miRNAs	Forward Primers (5′-3′)
bta-S18(F)	CACCGAGGATGAGGTGGA
bta-S18(R)	TATTGGCGTGGATTCTGC
bta-miR-196a	GCTGCGACCGTAGGTAGTTTCAT
bta-miR-205	TCCTTCATTCCACCGGAGTCTG
bta-miR-200b	GCTGACGGTGCTAATACTGCCT
bta-miR-223	CCTGTCAGTTTGTCAAATACCCCA
bta-miR-184	TGGACGGAGAACTGATAAGGGTAAA
bta-miR-1246	GAATGGATTTTTGGAGCAGGAA

Table S8. The primers used for qRT-PCR to validate the microarray test.

Gene	Forward Primers (5′-3′)	Reverse Primers (5′-3′)	Length (bp)	GenBank ID
β-actin	CATCCTGACCCTCAAGTA	CTCGTTGTAGAAGGTGTG	91	NM_173979.3
DGAT2	GTGTACTCTGCTTTCTCTTGA	ATGCTAATGTGACCCTAACAAT	117	NM_205793
FADS2	GGTACCCAATGGGACAAAGA	TAGTGGGTTAATTTCCAGATGC	131	NM_001083444
ACOX3	GCTGACTACTTGTAATTTGGGA	AAAGAATCGGACTGGCAT	113	NM_001103236
ALDH3A2	TGTTTCACTGCTGTTGTCAT	GCAGGAAACTTGAAGATTCG	105	NM_001101984
EHHADH	ATGAGATTGCTTCTTCCAGT	CGGCTGGGAATAACCTCTAA	100	NM_001075780
FASN	TAAGGTTCAAATTGCTGCGT	GAGCGAAGGAGAGATTTAATCA	134	NM_001012669
LPL	TGTGACTTGTTGTTGGCA	AGAGTTCCCAGGGCCATA	126	NM_001075120
SCD	TGCAGAAGTGGCTGGTATAAA	CTTCTACCCTCCATCAAATGTG	142	NM_173959
SLC27A6	TTAGGTGCCACTTGTGTGTTA	GCAAAGGTAGTGACAAAGTTC	117	NM_001101169
SREBF1	CAGGTGACTCAACTGTTCC	GCATCTGAGAACTCCTTGT	110	NM_001113302

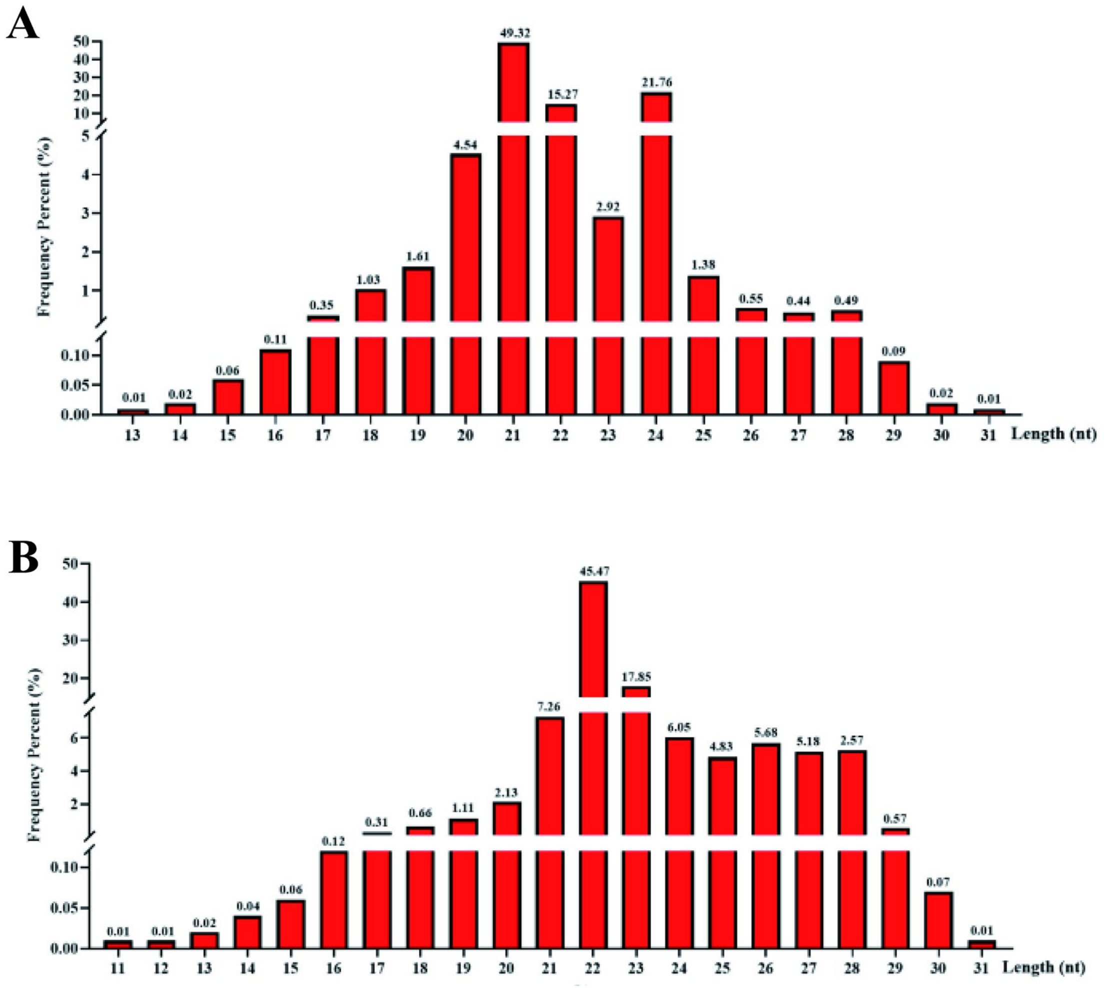

Figure S1. The length distribution of small RNAs in (A) control group and (B) *S. aureus*-inoculated group.

Integrative analysis of miRNA and mRNA expression profiles in mammary glands of Holstein cows artificially infected with *Staphylococcus aureus*

Xiaolong Wang[1†], Yongliang Fan[1†], Ziyin Han[1], Zaicheng Gong[2], Yalan Peng[2], Yongjiang Mao[1], Zhangping Yang[1, 3], Yi Yang[2, 3]*

[1] Yangzhou University College of Animal Science and Technology, Yangzhou, Jiangsu, China

[2] Jiangsu Co-innovation Center for the Prevention and Control of Important Animal Infectious Diseases and Zoonoses, Yangzhou University College of Veterinary Medicine, Yangzhou, Jiangsu 225009, China

[3] International Corporation Laboratory of Agriculture and Agricultural Products Safety, Yangzhou, Jiangsu, China

† These authors contributed equally to this study.

* Correspondence: Yi Yang, E-mail: yangyi@yzu.edu.cn
